# The use of telepsychiatry during COVID-19 and beyond

**DOI:** 10.1017/ipm.2020.54

**Published:** 2020-05-21

**Authors:** M. O’Brien, F. McNicholas

**Affiliations:** 1 Department of Psychiatry, CHI at Crumlin, Crumlin, Dublin 12, Ireland; 2 Department of Psychiatry, Lucena Clinic, Rathgar, Dublin 6, Ireland; 3 Department of Child and Adolescent Psychiatry, SMMS, UCD, Dublin 4, Ireland

**Keywords:** Coronavirus, COVID-19, mental health, pandemic, psychiatry, technology, telemedicine, telepsychiatry

## Abstract

The COVID-19 pandemic has disrupted the traditional practice of psychiatric assessment and treatment via face-to-face interaction. Telepsychiatry, the delivery of psychiatric care remotely through telecommunications technology, is an existing and under-utilised tool that may help to minimise disruption to patient care. Technological advancement is at a stage where it can facilitate widespread use of this practice; however, concerns that limited its expansion previously were not unfounded. This article discusses the use of telepsychiatry in the context of the COVID-19 pandemic.

## Introduction

The COVID-19 pandemic has presented the medical community with a sweeping collection of challenges. Given the nature and scale of the problem and the speed of its spread across the globe, no healthcare system has the necessary infrastructural resources to adequately cope with the evolving impact of this novel coronavirus. Moreover, the effects of this pandemic will likely be present for some considerable time and hence management initiatives that seek to overcome barriers to medical care caused by physical distancing measures must be sustainable over a protracted period. National healthcare systems are radically restructuring and reorganising their services with the intention of expeditiously diagnosing and treating those affected by the disease. Meanwhile, every effort is being made to retain and preserve existing service provision as non-COVID-19-related medical need persists. It is a source of concern that patients may become increasingly reluctant to present with other serious health problems due to fear of contracting the virus in a healthcare setting (Fagan, 2020). As well as the difficulties the pandemic is causing for patients, negative effects on the physical and mental well-being of healthcare staff can compromise the collective response. Reduced staffing numbers due to necessary self-isolation in confirmed, suspected or potential positive cases not only limits the capacity for the delivery of medical care but also places a greater strain on the remaining staff members. Resultant stress and burnout can insidiously erode productivity and ultimately impede patient care. Examination of the psychological impact on healthcare employees during the 2003 SARS outbreak found that approximately 10% of respondents experienced high levels of post-traumatic stress symptoms (Wu *et al.* 2009). There are already high rates of burnout among Irish hospital doctors across all specialties (Hayes *et al.* 2017) and within psychiatry in the child and adolescent mental health service (McNicholas *et al.* 2020). This, coupled with concerns that rates of job stress in Ireland may be higher than other European countries (Russell *et al.* 2018), increases the vulnerability of the workforce to widespread psychological sequelae resulting from this pandemic. The rapid and evolving changes to procedures and protocols in healthcare settings are a further burden on staff. Typically, healthcare systems introduce change in a slow and purposeful manner and only following extensive planning, training and pilot testing. The enforced response demanded by COVID-19 is radically different, as abrupt widespread changes to practice have had to be incorporated in a short period of time. These have impacted on every facet of healthcare activity, both in hospital and community settings.

In the context of growing healthcare demand, an overburdened healthcare system, a need to physically distance and a population who may view healthcare settings and personnel as potential sources of avoidable infection, clearly a different approach to healthcare engagement is required.

Hence, due consideration should be given to a tool that simultaneously enables us to connect and to distance at the same time – telemedicine.

## Telemedicine

Telemedicine refers to the use of telecommunication technology for the remote assessment and treatment of patients. Though the term is often used interchangeably with ‘telehealth’, the latter typically denotes a broader scope of healthcare services involving not only clinical assessment and treatment but also non-clinical activities such as patient education, provider training and administrative meetings. The use of telemedicine is not a new concept. It has evolved over time in parallel with developing technologies. An instance of its use was described in The Lancet in 1879, when an anonymous writer reported a case where a doctor successfully diagnosed a child over the telephone in the middle of the night (Bashshur & Shannon, 2009). One of the first instances of the use of television images for medical treatment occurred in the late 1950s when the Nebraska Psychiatric Institute established a closed-circuit television link with Norfolk State Hospital, allowing for consultations (Von Hafften, 2020). The beginning of manned space flight saw acceleration in the development of telemedicine as the American National Aeronautics and Space Administration (NASA) sought to manage the health of astronauts remotely (Bashshur & Shannon, 2009). Indeed, the advent of personal computers and the internet in the 1990s and 2000s has led to ever-increasing opportunities in the field. However, despite sufficient technological advancement, the widespread use of telemedicine has been slow to evolve (Smith & Gray, 2009; Barnett *et al.* 2018). Patient reticence, lack of regulatory framework and inherent limitations, such as the inability to physically examine, have all likely contributed to the low adoption rates.

## Telepsychiatry

Telepsychiatry is the term given to the application of telemedicine within the specialty of psychiatry. Telemental health and telepsychology are associated terms referring to the administration of psychological treatment or therapy via technological means. Compared to a more procedural-based specialty, psychiatry is relatively well suited to remote engagement and so is in a good position to transition to a telemedicine approach. The largely talk-based practicalities of psychiatry lend itself to the practice. Functionally, telepsychiatry typically involves an interaction between a psychiatrist and a patient via either telephone or videoconference. Research has indicated that telepsychiatry is comparable to face-to-face services in terms of reliability of clinical assessments and treatment outcome (Hubley *et al.* 2016). It has also been shown that it delivers high patient satisfaction among those suffering with movement disorders such as Parkinson’s disease (Seritan *et al.* 2019). There have even been suggestions that it may be preferable to in-person interaction in a subset of patients, including those with severe anxiety disorders (American Psychiatric Association, 2020). As always, caution must be exercised when interpreting such reports with due regard to patient population under review and particularly with regard to other factors such as socioeconomic status, computer literacy and age profiles. The use of telepsychiatry can also be expanded to facilitate group therapy. A systematic review of the evidence for telehealth group-based treatment found video teleconference groups to be feasible and to result in similar treatment outcomes to in-person groups (Gentry *et al.* 2019). However, the potential for small decreases in therapeutic alliances was noted. It was also suggested that additional research is needed to identify optimal methods of video teleconference group delivery to maximise clinical benefit and treatment outcomes.

Despite the promise shown, concerns that have limited the expansion of the more widespread application of telepsychiatry are not unfounded. Barriers to its use which have been identified include considerations about rapport, privacy, safety and technological limitations (Cowan *et al.* 2019).

## Prior use in disaster scenarios

Though the use of telepsychiatry, and indeed telemedicine more broadly, has been limited to date, their benefit in prior widespread emergencies has been documented. Telemedicine has previously been effectively used in simulated and real disaster scenarios (Simmons *et al.* 2008; Doarn *et al.* 2018; Uscher-Pines *et al.* 2018). It was utilised effectively in the aftermath of hurricane Ike which struck Texas in 2009, killing 82 people (Wicklund, 2017). Physicians from the Center for Telehealth Research and Policy at the University of Texas Medical Branch (UTMB) in Galveston noted that ‘Although there were significant disruptions to a majority of UTMB’s physical and operational infrastructures, its telemedicine services were able to resume near normal activities within the first week of the post-Ike recovery period, an unimaginable feat in the face of such remarkable devastation’. They also credited the ‘plasticity’ of the telemedicine programme. Telepsychiatry specifically has also been utilised in prior natural emergencies (Qadir *et al*. 2016) and is notably applicable in the context of the inevitable psychological sequelae that result. However, its use has previously only been considered when dealing with a relatively localised disaster, with help being administered remotely from other locations. The widespread nature of the current emergency is unique.

## Telemedicine in COVID-19

The patient-related advantages of the use of telemedicine prior to the outbreak of COVID-19 largely centred around convenience and efficiency. The service allows patients to dispense with travel to the hospital or clinic and also allows for more flexibility with securing appointments. Since the outbreak, the primary consideration has been one of safety and adherence to mandated physical distancing measures. Telemedicine provides a means to bring doctors and patients together without risking contamination. With regards to suspected cases of COVID-19, it allows for triage without healthcare worker exposure to the virus. Suspected cases can initially be assessed remotely, and investigation and treatment plans developed accordingly. In primary care settings, it allows for continued patient monitoring and assessment, as well as prescription of medications. It also facilitates the continuation of outpatient services across all medical specialties, though the limitations of reduced capacity to physically examine disproportionately affect some specialities more than others. With specific regard to psychiatry, the utilisation of a telemedicine approach facilitates the potential for the preservation of a large proportion of doctor–patient interactions. It allows for ongoing therapeutic work, especially when there is an established relationship between clinician and patient. However, there are some considerations which need to be taken into account.

## Clinical considerations

The initial consideration relates to the patient’s level of familiarity with the applicable technology and their ability to utilise it effectively and with ease. This method of engagement may be less suitable for certain cohorts of patients, such as those who have diminished cognitive capacity or are less technologically literate, for example. Equally, those with hearing difficulties, visual impairment or poor manual dexterity may struggle with the practicalities of its use. These limitations inherently disproportionately affect elderly patients, and unless they can be adequately supported in its use, the embrace of telepsychiatry should be tempered with cognisance of the potential for an imbalance in patients’ access to care. A similar imbalance may also be caused by the digital divide – the uneven distribution of access to required technology such as smartphones or high-speed internet. This divide is often affected by geographical or socioeconomic realities. It is prudent that clinicians take these factors into account and strive to ensure equitable access to care for all of their patients. This may involve reverting to face-to-face engagements where clinically indicated.

Beyond such logistical concerns, the important aspect of rapport needs to be considered, as some patients may find remote engagement to be a barrier to establishing a bond with their clinician. It might be more acceptable to some if they already have an established, trusting therapeutic relationship with their healthcare provider. In such circumstances, the telepsychiatry assessment is intended to supplement but not replace the more traditional face-to-face encounter.

In terms of securing the patient’s informed consent, the nature and purpose of the interaction must be fully explained in advance, together with the known limitations such as an inability to perform a physical examination or to identify other cues that might be relevant to making a diagnosis. The Irish Medical Council states in its ‘Guide to Professional Conduct and Ethics for Registered Medical Practitioners’ that an explanation should be made to patients that there are aspects of telemedicine that are different to traditional medical practice, including the inability to perform a physical examination (Medical Council Ireland, 2019). Though psychiatry may be less reliant on physical examination than other specialties, it is nevertheless a key component of a comprehensive assessment. Separate to the act of performing a structured physical examination, huge amounts of information are garnered from being in the physical presence of a patient. An odour of alcohol, loose-fitting clothes indicating weight loss and gait abnormalities are all examples of integral pieces of information that are lost with the use of telepsychiatry. Ultimately, this consideration needs to be factored into the overall clinical picture, and arrangement for physical examination and investigation made at the earliest convenience.

Concern has also been expressed about the ability to respond to psychiatric emergencies from a remote standpoint (Hubley *et al.* 2016). However, there are no published reports of instances of same related to telepsychiatry. In a circumstance where there is a likelihood of imminent harm to the patient or others, immediate contact should be made with An Garda Síochána, who may perform a welfare check and intervene in accordance with Section 12 of the Mental Health Act 2001, if applicable (Government of Ireland, 2001). Clinicians in secondary care often deal with complex clinical scenarios, and the introduction of telemedicine may require new strategies for managing certain clinical scenarios which could arise with this new method of assessment.

Matters of privacy and confidentiality are essential elements in all doctor–patient relationships. When conducting a remote interview, the interviewer cannot be certain that the person is in a secure and private setting. Other parties may be surreptitiously present but not within the range of the camera. The potential to influence the patient in terms of how he/she reports symptom burden and experiences may be influenced in such circumstances. It is important therefore that the interviewer seeks to establish precisely who is present when conducting a telemedicine interview. Depending on an individual’s social circumstances and housing situation, it may or may not be possible to secure a private location.

Another key aspect of comprehensive clinical care is involvement from various members of the multidisciplinary team. The nature of a one-on-one remote assessment by telepsychiatry may impact negatively on the conduct of a holistic multi-faceted review. It is important that the integral role of the full multidisciplinary team (MDT) is not overlooked due to a change in the modality of assessment and treatment.

## Record-keeping and indemnity considerations

Comprehensive and contemporary medical note-taking is a crucial component of the doctor–patient interaction. Medical records safeguard patients by allowing for continuity of care and offer medicolegal protection to doctors by accurately reflecting information present to them at the time of decision-making. Medical record practices for telepsychiatry are the same as for face-to-face contact. Before an assessment, access to the patient’s existing medical notes should be available and reviewed. After the engagement, prompt recording of appropriate medical notes should be made. There are however some additional points of information that should be documented in a telepsychiatry interaction. It should be clearly noted that the assessment was not conducted face-to-face and that the patient gave informed consent to proceed, cognisant of the strengths and limitations of this engagement. A record of the location of both the doctor and patient should be recorded. One particular indemnity consideration that has become pertinent with telepsychiatry is the circumstance of a patient seeking remote medical assessment and treatment when they are outside Ireland. In this case, the initial course of action should involve talking to the patient and assessing the situation (Hendry, 2020). A recommendation should then be made to the patient about the most appropriate alternative route for assistance, which may involve seeking local medical care. Another consideration for clinicians is the capacity for patients to record telepsychiatry interactions. A prior cross-sectional survey completed in the United Kingdom found that 15% of respondents had secretly recorded a clinic visit (Elwyn *et al.* 2017). The United Kingdom’s Medical Defence Union advises that patients do not require clinician’s permission to record interactions and that such recordings, even if taken covertly, have been admitted as evidence of wrongdoing in court (Zach, 2014). This behaviour is clearly not limited to telepsychiatry and can and does happen in face-to-face consultations (Elwyn *et al.* 2015). Furthermore, it has been viewed as a positive component of therapy, facilitating patient empowerment and enhancing their care experience, with the ability to own, replay, share and reflect on the clinical encounter. This might raise concern for the clinician, especially if such recordings are conducted covertly and motivated by a desire to obtain evidence of inadequate or unsatisfactory care. Ultimately, the optimum circumstance is for openness and transparency when patients seek to record interactions, something which doctors should strive to facilitate.

## Data concerns/security

All healthcare records are confidential, and every effort must be made to ensure that all personal data are appropriately recorded and stored. All personal data must be processed and stored strictly in conformity with the General Data Protection Regulation (GDPR) legislation which in Ireland came in to force in May 2018. This legal framework defines and regulates the ways in which personal data are collected and used. A detailed description of GDPR legislation is beyond the scope of this paper, but it includes several key principles encompassing lawfulness, transparency and fairness, purpose limitation, data minimisation, accuracy, storage limitation and integrity and confidentiality (Data Protection Commission, 2018). Personal data include information concerning a living individual who can be identified whether directly or indirectly. Healthcare professionals are required to collect a high volume of personal data and are in breach of professional guidelines if they fail to maintain proper, accurate and relevant healthcare records. Therefore, the security of the IT platform when providing telepsychiatry services is of paramount importance. It is best practice to use a health system approved secure network which incorporates appropriate measures to ensure an optimal level of security. As referenced above, prior to embarking on a telemedicine interview in which personal data are divulged, consent must be obtained and recorded as such. Consent must be freely given, fully informed and clearly reflect the patient’s wishes. Failure to object or refuse in itself does not constitute consent, and all consent may subsequently be withdrawn in both face-to-face and remote treatment circumstances.

## Practical considerations

For many healthcare professionals, telepsychiatry represents a novel way of engaging with patients and families. Individual practitioners may have varying levels of comfort when using this technology. A basic level of IT literacy is required by the clinician and access to the necessary devices and reliable high-speed internet is of course essential. Clinicians will need to develop the relevant skills and competencies necessary to conduct assessments using telepsychiatry, with ‘webside manner’, the telemedicine equivalent of bedside manner, being a key component of the professional telepsychiatry interaction. Two of the key components of webside manner are audibility and visibility. Audibility can be maintained through proximity to the microphone, and reduction of ambient noise and visibility can be enhanced by appropriate lighting and by avoiding positioning a window in the background. Furthermore, an uncluttered and simple background minimises the potential for distraction, and every effort should be made to reduce the potential for disturbance or interruption of the session. Establishing ‘virtual eye contact’ by looking directly into the camera, using the person’s name often, ensuring adequate time for them to finish their contributions and making frequent use of non-verbal encouragements may mitigate against the lack of physical presence (McConnochie, 2019).

## Beyond COVID-19

While telepsychiatry is a particularly apt solution to the problems posed by physical distancing measures during this pandemic, its scope for benefit existed before the current crisis and will continue long after it. The adoption of its use has been dramatically accelerated due to unprecedented need, though the move towards technology-mediated medical intervention is long established. In 2018, the European Commission estimated that the global telemedicine market would reach €37 billion by 2021 (O’Brien, 2020). This is likely to be surpassed when the current spike in use is factored. It is crucial that this current era of increased use of telemedicine be seen as fertile ground for research, providing guidance for future direction. Increased prevalence of use, shored up by this research, will likely add to the legitimacy of telemedicine as a component of delivery of care. Further reassurance may be given to clinicians through appropriately developed legal and regulatory framework. The incorporation of telemedicine-related teaching into medical school curricula would also increase homogeneity and consistency of approach. It is prudent that this inevitable development be coordinated with purposeful and proactive measures on both local and organisational levels, with a view to securing functional integration long beyond this pandemic. These measures are by their very nature disruptive as they inherently involve the restructure of systems and therefore effective change management strategies should be employed to ensure user adoption is sustained. Technology is no longer the limiting factor, so focus now needs to be shifted towards financial, systematic and cultural considerations. The widespread reliance on telemedicine during this crisis will likely emphasise its importance and aid in building trust in it as a viable treatment tool. This in turn will ultimately lead to lasting evolution in medical and psychiatric practice.
